# Comparative Evaluation of New Needleless Local Anesthetic System (INJEX) and Conventional Syringe Needle Technique during Pulpotomy Treatment: A Randomized Clinical Trial

**DOI:** 10.3390/children11050514

**Published:** 2024-04-25

**Authors:** Satish Vishwanathaiah, Nassreen H. Albar, Fatemah Tawfeg Abkar Alraghi, Noor Eissa Mousa Jaferi, Ishraq Abdullah Mohammed Tumayhi, Suman Panda, Fatima Ali Hassan Khormi, Atlal Hassan Hussain Jaafari, Zahra Ahmed Ibrahim Abiri, Prabhadevi C. Maganur

**Affiliations:** 1 Division of Pediatric Dentistry, Department of Preventive Dental Sciences, College of Dentistry, Jazan University, Jazan 45142, Saudi Arabia; svishwanathaiah@jazanu.edu.sa (S.V.); supanda@jazanu.edu.sa (S.P.); 2 Restorative Dentistry Department, College of Dentistry, Jazan University, Jazan 45142, Saudi Arabia; nalbar@jazanu.edu.sa; 3 Dental School, College of Dentistry, Jazan University, Jazan 45142, Saudi Arabia; 201706832@stu.jazanu.edu.sa (F.T.A.A.); 201600058@stu.jazanu.edu.sa (N.E.M.J.); 201600031@stu.jazanu.edu.sa (I.A.M.T.); 201706377@stu.jazanu.edu.sa (F.A.H.K.); 201601328@stu.jazanu.edu.sa (A.H.H.J.); 201500752@stu.jazanu.edu.sa (Z.A.I.A.)

**Keywords:** behavior, pain, INJEX, traditional system, conventional system, FBRS, Wong–Baker scale, FLACC

## Abstract

Background: The dreaded sensation of pain in the dental chair has a significant impact on children’s behavior. This study aimed to compare and contrast the perception of pain and patient behavior between the use of INJEX and the conventional syringe needle technique during pulpotomy among children. Methods: A randomized clinical trial was designed and conducted among pediatric dentistry patients aged 6–12 years old. Fifty-eight children were divided into two groups, conventional syringe needle and INJEX, using simple randomization method applying the sequentially numbered, opaque, sealed envelope method of allocation concealment. Anesthesia was administered to the groups as local infiltration by a single operator following routine behavior guidance techniques. After 3 min, pulpotomy was performed using the standard protocol. The Face, Legs, Activity, Cry, Consolability (FLACC) scale and Wong–Baker FACES Pain Rating Scale (WBS) were used to assess the intensity of pain, while the Frankl behavior rating scale (FBRS) was used to assess the children’s behavior. Results: During anesthesia, most of the participants in the INJEX group (median = 3) had higher FBRS scores compared with the conventional syringe needle group (median = 2), and the difference was very highly significant (*p*-value < 0.001). Analyzing the FLACC scores during local anesthesia administration revealed a high statistical significance (*p*-value < 0.01) across the two groups. A very high statistically significant difference (*p*-values < 0.001) with higher WBS scores for pain intensity was seen in the group using conventional syringe needles. Conclusions: INJEX administration significantly reduced the intensity of pain experienced by the children and helped maintain a positive attitude among them during pulpotomy. It provided a positive and comfortable experience for both the child and the practitioner. Therefore, it can serve as an excellent alternative to conventional needle anesthesia.

## 1. Introduction

Local anesthesia (LA) has been a core component of dentistry since its inception and remains necessary despite advancements in techniques and tools. It is considered an effective method for managing pain during invasive dental procedures [1]. LA acts by blocking the rapid influx of sodium ions within neural fibers, which is necessary for the generation and propagation of neuron impulses to sense pain [2]. This technique not only aids in the prevention of pain during dental procedures but also helps in nurturing trust between dentist and patient, reducing fear and anxiety and endorsing a positive attitude toward dental care [3].

The primary issues that hinder patients from obtaining regular dental treatment are nervousness and a profound fear of pain [4]. The impact of pain on a child’s behavior is an important element to consider; thus, it is crucial to comprehend the pain experienced by a child during a procedure. As children have difficulty verbalizing pain due to developmental, cognitive, or communication barriers, numerous scales are used. The Face, Legs, Activity, Cry, Consolability (FLACC) scale, which is an observational pain scale, and the Wong–Baker FACES Pain Rating Scale (WBS), which is a self-assessment tool/subjective scale, are commonly used in the pediatric population to assess pain in infants and children who are unable to verbally express their pain [5,6,7]. Studies have found some positive correlations between these scales. The next factor to consider is behavior, and the most commonly used scale to classify behavior based on attitude and cooperation is the Frankl behavior rating scale (FBRS), which comprises Category 1 (definitely negative), Category 2 (negative), Category 3 (positive), and Category 4 (definitely positive). This score is determined by the treating clinician [8].

A large number of individuals suffer from “needle phobia” or blenophobia, which is the intense fear of needles [9]. The fear of receiving a dental injection can be frightening for both children and adults, mainly because of pain during the injection [1]. Administering LA to children without causing pain has always been a challenging aspect of dental treatment. Effectively managing pain and addressing the fear associated with local anesthesia administration can reduce a child’s worries to a certain extent and in turn help in cultivating an overall positive behavior toward dental treatment [10]. Children often find the injection of LA with a regular needle syringe to be unpleasant, causing fear and anxiety. This can result in patients wanting to terminate or delay their treatment [4]. Research has shown that there is a connection between anxiety, fear, and the perception of pain. Feeling anxious and fearful can lead to increased stress levels, causing a lower threshold for feeling pain [11]. Although dentists cannot control these fears, certain aspects of treatment can be adjusted to improve children’s comfort.

Although needles and anesthetic agents have seen a remarkable transformation in quality and design over the past few decades, the process of administering LA remains unchanged [12]. Unfortunately, the conventional method of using a needle brings about discomfort during the piercing and injecting phases. This discomfort may stem from the mishandling of the syringe, the application of excessive pressure on the needle, and the rapid injection of copious amounts of solution [4]. Various methods have been cited in the literature to alleviate discomfort during LA. These include applying LA before the injection [13], using computerized injection systems [14], manually adjusting the injection speed [15], and employing needleless jet injection devices [16].

Needleless jet injection devices, which use high-velocity fluid to penetrate tissues, have been proposed as a needle-free alternative to conventional local anesthesia administration. These jet injectors operate by propelling a small amount of medication through a narrow opening using compressed gas or a spring. The key advantage of this method is that it eliminates the pain and fear associated with needles and local anesthesia administration [10,17]. Dental jet injectors, such as Syrijet (Keystone Industries, USA) and Panjet (Wright Health Group Limited, UK), have been adopted since the 1970s and can effectively anesthetize the target tissue while improving patient comfort [18]. Currently, newer jet injection systems, including Madajet (Mada Medical Products, USA) [19], INJEX (INJEX Pharma AG, Germany) [20], and Comfort-in (Mika Medical Co., Republic of Korea) [21], are being utilized in dentistry to administer LA. Our study was undertaken to compare and contrast the perception of pain and patient behavior between the use of INJEX and the conventional syringe needle technique during pulpotomy among children.

## 2. Materials and Methods

### 2.1. Study Design, Ethical Clearance, and Informed Consent

**Figure 1:**
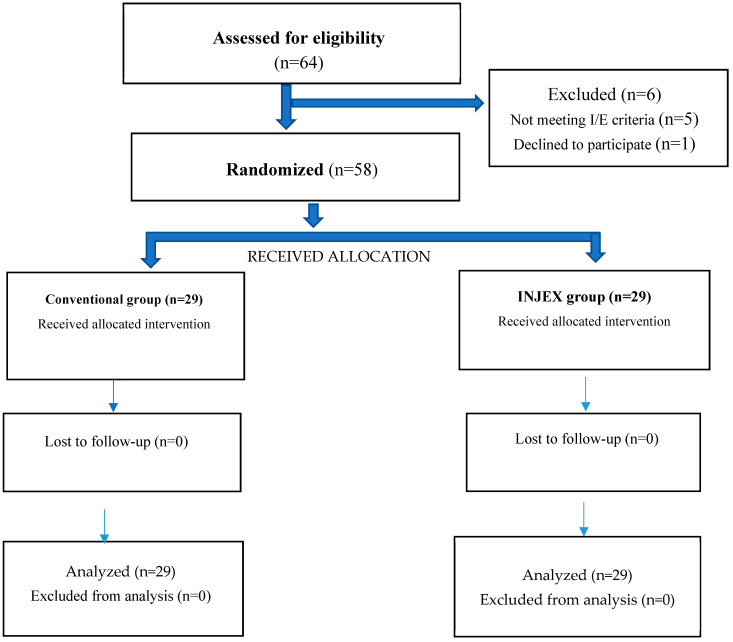
Schematic representation of the study design (Consolidated Standards of Reporting Trials flow diagram).

This randomized, single-blinded, controlled trial was assessed, analyzed, and approved by the Institutional Human Ethical Committee of the College of Dentistry, Jazan University, with the registration number REC-44/07/503. The experimental design followed the Consolidated Standards of Reporting Trials (CONSORT) guidelines (see Figure 1). The complete protocol of our study, including the finer nuances, was explained to the parent or guardian, and only after obtaining their signed written informed consent was the participant included in the study. This study was performed according to the ethical standards of the Declaration of Helsinki (1964) and its subsequent amendments.

### 2.2. Sample Size Estimation

The sample size was estimated according to the given formula with a difference in mean FLACC scores of 1.2 and a pooled standard deviation of 1.89, which were obtained from a previous study [22].

$$\mathrm{Sample}\;\mathrm{Size}\;(n)=\frac{2{S_{p}}^{2}{\left[Z_{1-\alpha /2}+Z_{1-\beta }\right]}^{2}}{{{µ}_{d}}^{2}},$$

where 

Z_1−α/2_ = 1.96 for 95% confidence interval;

Z_1−β_ = 0.84 for 80% power;

S_p_^2^ = pooled standard deviation; 

µ_d_ = 1.2 (difference in means between the groups).

By substituting these values, the sample size was estimated to be 21. Twenty percent of the estimated sample size was added to compensate for any sampling losses. Hence, the final sample size included 29 participants in each group (see Figure 1).

### 2.3. Participant Selection

#### 2.3.1. Inclusion Criteria

Initially, children aged between 6 and 12 years old who reported to the outpatient section of the pediatric dental department, College of Dentistry, Jazan University, were selected. The inclusion criteria involved children who exhibited overall physical and mental well-being and who did not have any complicated medical history. In particular, the children who were categorized under Category 3 and Category 4 based on Wright’s modification [8] of the FBRS during the initial examination and intra-oral periapical radiographs were selected for the study. Another integral inclusion criterion involved the presence of a deep carious lesion in the primary maxillary or mandibular molar that required pulpotomy. The parents of all the participants chosen for the study provided written informed consent and displayed a willingness to participate in the randomized controlled trial.

#### 2.3.2. Exclusion Criteria

Children below the age of 6, those with symptoms of irreversible pulpitis and dentoalveolar abscess, those categorized under Category 1 and Category 2 according to Wright’s modification of the FBRS [8] during the initial examination, and medically or mentally compromised children were excluded from the study. 

#### 2.3.3. Randomization and Allocation Concealment

The children were divided into two groups, Group I (conventional needle syringe) or Group II (INJEX), using a simple randomization method and applying the sequentially numbered, opaque, sealed envelope method of allocation concealment.

#### 2.3.4. Study Groups 

**Figure 2:**
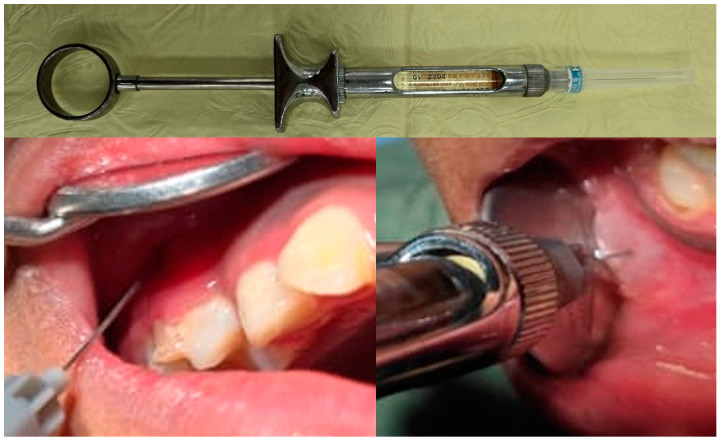
Group I (conventional syringe).

Group I—All participants in Group I were subjected to the conventional needle syringe system. The local infiltration of Scandicaine 2% Speciale (mepivacaine hydrochloride and adrenaline) was administered using a conventional needle syringe with a 27-gauge needle (Hogen Spitze, C-K Dental Ind. Co., Ltd.; Bucheon, Gyeonggi, Republic of Korea) [Figure 2].

**Figure 3:**
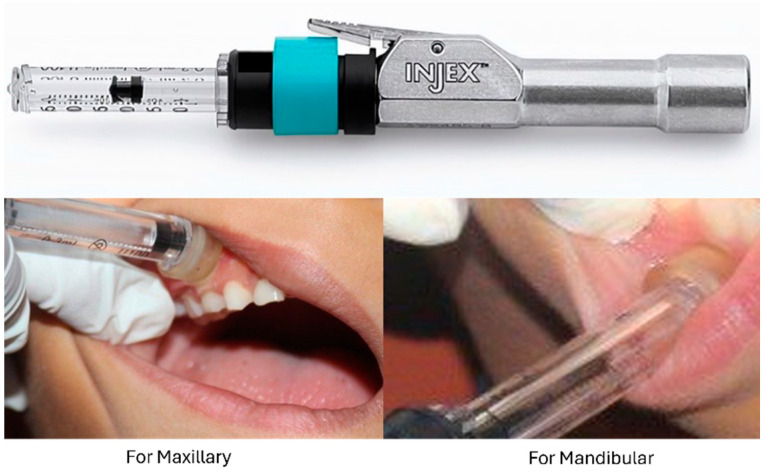
Group II (INJEX).

Group II—The participants in this group experienced the needle-free injection system INJEX. The INJEX system’s components include an injector that administers the injection, a single-use sterile plastic ampoule attached to the injector that holds the medication (0.3 mL of Scandicaine 2% Speciale), an adaptor that facilitates the transfer of the drug into the ampoule, and a reset box that resets the device to proceed with the injection process. The injector must be reset before each use. In this process, the injector is placed inside the reset box and sealed to initiate a lever mechanism that compresses the spring within the injector for recharging [12]. The prepared injector is securely positioned on the mucosa, and with a brief press on the trigger, the medication is delivered. A standard pressure of 3000 psi is used to administer the injection, and the medication is delivered to the cutaneous/subcutaneous tissue at a depth of 5–8 mm [Figure 3].

### 2.4. Intervention Procedure

To avoid any operator-related bias, a single operator handled the entire anesthesia protocol for all the participants in the trial. All the treatment-relevant equipment and treatment protocols were introduced and explained using the tell–show–do technique, and the injection was described using appropriate euphemisms. The treated area at the injection site was initially cleansed using sterile dry gauze. A minimal amount of topical anesthetic (benzocaine 20%, Lakewood, NJ, USA) was applied, remaining in position for a minimum of 1 min. Based on the group to which they were allotted, LA was delivered after the application of topical anesthesia. After a standard waiting time of 3 min for the initiation of the anesthesia, pulpotomy was performed. 

### 2.5. Outcomes

One investigator measured the primary outcomes, such as the FLACC [23] and WBS [24] scores. Another investigator measured the secondary outcomes, including the time required to deliver the anesthesia, FBRS score [25], and pulse rate.

### 2.6. Primary Outcomes

#### 2.6.1. Face, Legs, Activity, Cry, Consolability Scale

The FLACC scale was the objective pain assessment tool used during LA administration. It evaluates five domains of behavior to assess pain intensity: Face—assessed facial expressions, such as grimacing and frowning that are indicative of pain; Legs—leg movement or tension, considering signs such as restlessness or tenseness; Activity—overall body activity, including general restlessness or the inability to stay still; Cry—vocal expressions of pain, such as crying or vocalizations; and Consolability—the ease with which the child can be comforted or consoled. A score of 0 or 2 is given for each domain, with 0 indicating no pain or distress and 2 indicating the highest level of pain or distress. The total FLACC score ranges between 0 and 10 and is obtained by adding the individual scores from each domain. Lower scores signify lower pain intensity levels, while higher scores indicate severe pain levels [23]. 

#### 2.6.2. Wong–Baker FACES Pain Rating Scale

The WBS was the subjective pain assessment tool used in this study. The scale is composed of six facial expressions, each assigned with a numerical value ranging from 0 to 10 to denote pain intensity. The scale’s scores are used to categorize pain levels, with a value between 0 and 4 indicating mild pain, 4–6 denoting moderate pain, 6–8 signifying severe pain, and 8–10 representing unbearable pain. In both groups, the children were instructed to assess their pain intensity by selecting the most applicable statement on the WBS at four time points: before injection, immediately after injection, during treatment, and after treatment [24].

### 2.7. Secondary Outcome

#### 2.7.1. Time of Local Anesthesia Administration

The time required for the administration of the LA solution was documented. A third investigator recorded the total time from the time the topical anesthetic was applied up to the time the syringe/INJEX was removed from the participant’s oral cavity using a stopwatch.

#### 2.7.2. Frankl Behavior Rating Scale [25]

Each child’s behavior was assessed at different stages of the dental procedure using Wright’s modification of the FBRS. The FBRS is commonly used during children’s dental procedures and provides a structured way to evaluate their cooperation and responses. In our study, the children’s behavior was evaluated at various stages of the dental procedure, including intra-oral examination, radiography, application of topical anesthetic (grouped as “before” values), local anesthetic administration, and restoration (categorized as “after” values) [25].

#### 2.7.3. Pulse Rate

The pulse rate was employed as an anxiety indicator, as stress or anxiety can increase it. Pulse rate measurements were documented using a pulse oximeter device (Dr Trust Pulse Oximeter, Nureca Limited, India) affixed to the left index finger. The results were obtained in a very short time in relation to the physiological parameters. The data were gathered during a 15 min interval preceding LA administration, with fluctuations noted within that timeframe to determine the mean. Additionally, pulse rate readings during the LA injection and the 1 min post-injection period were independently recorded, and their averages were computed as “during” and “after” values, respectively. A third person who was not involved in the study and unaware of the protocol of the anesthesia procedure measured these secondary outcomes.

### 2.8. Statistical Analysis

The statistical analysis was conducted using a standard statistical package (IBM Corp. Released 2011. IBM SPSS Statistics for Windows, Version 20.0. Armonk, NY, USA). Data normality was assessed using the Shapiro–Wilk test. The chi-square test was used to compare the distribution of the participants between the two groups based on age, gender, and accompanying person. The intergroup comparisons of the metric and ordinal data were carried out using an unpaired *t*-test and the Mann–Whitney test, respectively. The intragroup comparisons of the metric and ordinal data were performed using a paired *t*-test and the Wilcoxon signed-rank test, respectively.

## 3. Results

The distribution of the participants between Groups I and II in relation to age and gender is described in Table 1. A statistically significant difference (*p*-value = 0.007) was observed for the distribution of the participants in relation to age, where the majority belonged to the age category of 6–9 years old in both groups (32.75%, 25.8%). 

A comparison of the time required for LA administration among the two groups showed a very highly significant statistical difference (*p*-value < 0.001), with conventional needle injection taking longer to complete (1.3 ± 0.39 min). A statistically significant difference was not observed for the pulse rates of the two groups, as shown in Table 2.

Upon analyzing the ordinal parameters, a significant difference was not observed for the FBRS scores before and after anesthesia, and most of the participants had a median score of 3 in both groups. During anesthesia, most of the participants in the INJEX group (median = 3) reported higher FBRS scores compared with the conventional syringe needle group (median = 2); the difference was found to be very highly significant (*p*-value < 0.001). Analyzing the FLACC scores during LA administration revealed a high statistical significance (*p*-value < 0.01) across the two groups. The median FLACC score in the conventional syringe group was 7 (4,11) and 2 (0,4) in the INJEX group. A very highly significant statistical difference (*p*-value < 0.001) was obtained for the WBS scores immediately after the local anesthesia administration between the groups, with higher scores [4 (2,6)] observed for the conventional syringe needle group, as shown in Table 3.

Table 4 and Table 5 depict the intragroup comparisons of the pulse rates, FBRS scores, and WBS scores before, during, and after LA administration between the conventional syringe and INJEX groups. A statistically significant difference in the pulse rates recorded was obtained (before versus during and during versus after) in the conventional group. A statistically significant difference was observed for the FBRS scores (before versus during and during versus after) in the conventional group, whereas in the INJEX group, the difference was significant only for one comparison (before versus during). Significant differences in the WBS scores were seen for all the values reported (immediately after injection and before, during, and at the end of treatment) in the conventional syringe group. In the INJEX group, statistical significance was obtained for the comparison between before versus immediately after injection and immediately after injection versus at the end of treatment.

## 4. Discussion

The fear of local anesthesia administration is one of the key factors fueling anxiety in young children during dental procedures. It holds significant sway over their emotions and often causes distress and unease. A child’s encounter with painless and fearless dental care will immensely enhance their ease during future visits to the dentist. Fear or anxiety related to dental procedures poses a substantial obstacle to obtaining routine dental care and impacts approximately 9% of the world’s population [26,27]. This leads to a high number of individuals avoiding dental treatment and neglecting regular check-ups, resulting in unfavorable oral health outcomes and adverse effects on overall health. Dental anxiety frequently stems from negative prior encounters with dental procedures, with LA administration through local anesthesia administration being a key factor in generating anxiety [28,29]. This is noteworthy because the proper delivery of LA can render dental treatments painless and comfortable for patients. However, it paradoxically becomes the primary source of anxiety for individuals with blenophobia. Especially among children, the sight of a needle during LA administration often induces more fear than the actual dental treatment [18].

Jet injection technology uses mechanical energy to generate sufficient pressure to push a liquid medication through a very small opening and into the subcutaneous tissue without utilizing a needle [30,31]. This needle-free method of administering anesthesia has several benefits, including a painless injection, minimal tissue damage, faster administration, and quicker absorption of the drug into the tissues compared with conventional needle delivery [18]. To the best of our knowledge, our study is the first of its kind to compare pain perception and patient behavior during pulpotomy between needleless and conventional LA systems. The intensity of pain experienced was assessed subjectively using the WBS, and a significant discrepancy in pain scores was seen immediately after LA administration. The needle group reported the highest pain rating of 4 (excruciating), whereas the children in the INJEX group reported zero pain during LA administration, showcasing the exceptional effectiveness of INJEX in alleviating injection pain. 

The impressive efficacy of the painless INJEX system was also apparent when examining the FLACC pain scale. Among the group of children who received conventional LA, the highest observed pain reached the maximum score of 7. In stark contrast, the INJEX group experienced far less discomfort, with a commendably low pain score of only 2. These compelling findings strongly suggested that the level of pain experienced during LA administration using the conventional method was significantly higher in comparison with INJEX. Deepak et al. [22] found that children who were randomized to receive a computer-controlled injection experienced significantly less pain during infiltration compared with the conventional system, which was similar to our findings. 

Evaluating pain through the WBS scale, the maximum score among the group who received conventional LA was 4, while for INJEX, it was 1 soon after LA administration. Our results did not align with Altan et al.’s findings [32], where needle-free systems and the dental needle method yielded no significant difference in pain perception during and after pulpotomy treatment.

The positive impact of the painless system on children’s behavior observed in our research was clearly reflected in the FBRS ratings. Initially, most of the children in both groups were rated as positive, but only those in the INJEX group maintained positive behavior throughout the procedure. This may be attributed to the appearance of the injector, which captured the children’s attention. Unlike conventional syringes, the hand pieces of the system have a different shape that can be more readily accepted by young children [22].

In the realm of dental literature, a lively debate exists surrounding the question of which needle-free approach yields a lesser degree of discomfort compared with conventional dental needle anesthesia. Agreement on this matter remains elusive, leaving us in a state of uncertainty regarding the most effective pain-free injection method. A study by Atlan et al. involving 100 children between the ages of 3 and 12 found that the Madajet XL needle-free system significantly decreased pain perception [32]. Ocak et al. [33] found that the INJEX jet injection system caused less discomfort during LA compared with the conventional dental injector method. By contrast, Oliveira et al. [29] observed no difference in pain perception between the needle-free injection system Comfort-In™ and the conventional dental injection in adults undergoing anesthesia. Lautenbacher et al. [34] compared the pain levels reported during conventional dental local anesthesia administration and INJEX administration involving 87 children. Their findings revealed that more pain was associated with the jet injection system, as the feeling of sudden pressure and the popping sound during the jet injection may have led to a fear response or misinterpretation of the pressure as pain [10,35].

The effectiveness of jet injection systems in providing pulpal anesthesia is also a subject of debate in the scientific literature. While these systems are generally effective in delivering soft tissue anesthesia, the administration of pulpal anesthesia for lateral maxillary teeth using the Syrijet system was less successful in 13% of patients in Ocak et al.’s study [33]. Out of 87 treatment procedures that were attempted after using INJEX in children, an extra injection was needed to achieve the necessary level of anesthesia in 80.5% of Arapostathis et al.’s study cases [20]. In our study, no extra injection was needed for maxillary or mandibular teeth, and pulpal anesthesia was effective for primary teeth.

Altan et al. [32] found that the onset time of the anesthetic effect was shorter with the Comfort-In™ injection system compared with the dental needle method, but the total anesthesia duration was higher with the dental needle method. A rapid decrease in the anesthesia’s effectiveness in the needle-free system may explain the notable increase in pain scores during pulpotomy, which was not on par with our study, where needleless anesthesia resulted in superior pain reduction. 

A needless injection can be swiftly delivered, taking no more than a fraction of a second [36]. Similarly, in our study, the total time taken for LA administration in the conventional group was three times more than in the INJEX group. Jet injection is preferred over needle-free systems for complicated surgical procedures or extractions, as illustrated by Theocharidou et al. [21], who used 0.3 mL of articaine 4%, and Oliveira et al. [29], who used 1 mL of lidocaine 2%. Both reported a significant reduction in the efficiency of the anesthesia 15 min after administration. It has been demonstrated in the literature that needle-free systems offer a proficient means of delivering anesthesia for various restorative dental procedures, such as Class I and Class II fillings, along with vital pulp [37]. 

Based on our findings, using 0.3 mL of Medicaine 2% (mepivacaine hydrochloride and epinephrine bitartrate) with INJEX for pulpotomy treatment showed better results compared with the dental needle method. In addition, notable disparities were observed in the effectiveness of the two injection methods, with the INJEX system surpassing the conventional approach in achieving deeper anesthesia, as demonstrated by the absence of any supplementary anesthesia requirements for the patients. 

Compared with other pressure anesthesia devices, the delivery of INJEX takes a 45° angle with the gingiva for optimal positioning. This means easier and more efficient administration, complete contact with the gingiva, minimal pressure, and no unpleasant taste or leakage. However, it is worth noting that the INJEX system does come at a higher cost compared with conventional needles. The administration of medication through a needle requires a delicate and vigilant approach due to its inherent risks and potential for harm. Proper needle handling techniques, such as maintaining the correct angle, are crucial to avoid causing severe damage. Conversely, needleless administration relies on the application of pressure, thus reducing the need for such precautions [1]. 

The standout aspect of our study lay in the careful selection of comparable gauge needles for the INJEX and conventional methods, which added credibility to the findings. The study’s main advantage was its employment of universally recognized pain scales, which enabled objective and subjective evaluations of pain with great accuracy. In addition, the trial was carried out by an expert, erasing any variances in operational efficiency. 

In this research, we did not attempt to quantify the postoperative pain levels of the children in both groups. Nevertheless, they were recalled, meticulously assessed, and bestowed with the requisite interventions in a timely and appropriate manner. Notably, our study only focused on single-needle gauge usage and did not include children with negative behavior. Moreover, it should be acknowledged that the difference in the efficacies of INJEX and conventional needle anesthesia in relation to the dental arch was not explored within the confines of this study. These limitations should be taken into account when applying our findings to the broader population. Further investigations should also be pursued to evaluate the utilization and effectiveness of needle-free local anesthesia administration for other treatment modalities in dentistry. 

## 5. Conclusions

INJEX administration can significantly reduce the intensity of pain experienced by children and help to maintain a positive attitude during pulpotomy. Injex was found to be superior in terms of the time required to deliver as well as the volume of anesthesia required for the procedure. Therefore, INJEX can serve as a viable substitute for traditional methods of administering anesthetic to pediatric patients. 

## Figures and Tables

**Table 1:** Distribution of study participants in relation to age and gender.

SI No.	Parameter	Conventional Syringe Needle n (%)	INJEX n (%)	*p*-Value
1	Age			0.007 **
	6–9 years old	19 (32.75)	15 (25.8)	
	9–12 years old	10 (17.24)	14 (24.13)	
2	Gender			0.189
	Male	12 (20.68)	18 (31.03)	
	Female	17 (29.31)	11 (18.96)	

** Significant at the 0.01 level.

**Table 2:** Comparison of various parameters (metric data) during local anesthesia administration.

SI No.	Parameter	Conventional Syringe Needle (Mean ± SD)	INJEX (Mean ± SD)	*p*-Value
1	Time required for local anesthesia administration in minutes	1.3 ± 0.39	0.34 ± 0.084	<0.001 ***
2	Pulse rate			
	Before administration of local anesthesia	92.57 ± 11.3	94.14 ± 10.27	0.583
	During administration of local anesthesia	102.93 ± 16.51	95.48 ± 13.94	0.0687
	1 min after local anesthesia administration	95.79 ± 13.23	95.03 ± 10.67	0.811

*** Significant at the 0.001 level.

**Table 3:** Comparison of various parameters (ordinal data) during local anesthesia administration.

SI No.	Parameter	Conventional Syringe Needle [Median (Q1,Q3)]	INJEX [Median (Q1,Q3)]	*p*-Value
1	FBRS			
	Before anesthesia	3 (3,4)	4 (3,4)	0.177
	During anesthesia	2 (2,3)	3 (3,4)	<0.001 ***
	After anesthesia	3 (3,4)	3 (3,4)	0.184
2	FLACC scale	7( 4,10)	2 (0,4)	0.005 **
3	WBS			
	Before procedure	1 (0,2)	1 (0,0)	<0.326
	Immediately after local anesthesia administration	4 (2,6)	0 (0,2)	<0.001 ***
	During treatment	0 (0,2)	0 (0,2)	0.603
	At the end of treatment	0 (0,0)	0 (0,0)	0.542

** Significant at the 0.01 level. *** Significant at the 0.001 level. FBRS = Frankl behavior rating scale; FLACC = Face, Legs, Activity, Cry, Consolability; WBS = Wong–Baker FACES Pain Rating Scale.

**Table 4:** Intra-group comparison of pulse rate and Frankl behavior rating scale during local anesthesia administration.

SI No.	Parameter	Group	Comparison	Mean ± SD/ Median (Q1,Q3)	*p*-Value
1.	Pulse rate	Conventional syringe needle	Before versus during	92.57 ± 11.3 102.93 ± 16.51	0.002 **
			Before versus after	92.57 ± 11.3 95.79 ± 13.23	0.289
			During versus after	102.93 ± 16.51 95.79 ± 13.23	<0.001 ***
		INJEX	Before versus during	94.14 ± 10.27 95.48 ± 13.94	0.449
			Before versus after	94.14 ± 10.27 95.03 ± 10.67	0.362
			During versus after	95.48 ± 13.94 95.03 ± 10.67	0.727
2.	FBRS	Conventional syringe needle	Before versus during	3 (3,4) 2 (2,3)	<0.001 ***
			Before versus after	3 (3,4) 3 (3,4)	0.06
			During versus after	2 (2,3) 3 (3,4)	<0.001 ***
		INJEX	Before versus during	4 (3,4) 3 (2,3)	0.007 ***
			Before versus after	4 (3,4) 3 (3,4)	0.145
			During versus after	3 (2,3) 3 (3,4)	0.437

** Significant at the 0.01 level. *** Significant at the 0.001 level. FBRS = Frankl behavior rating scale.

**Table 5:** Intra-group comparison of Wong–Baker FACES Pain Rating Scale during local anesthesia administration.

SI No.	Parameters	Group	Comparison	Median (Q1,Q3)	*p*-Value
1.	WBS	Conventional syringe needle	Before versus immediately after local anesthesia administration	1 (0,2) 4 (2,6)	<0.001 ***
			Before versus during treatment	1 (0,2) 0 (0,2)	0.005 **
			Before versus at the end of treatment	1 (0,2) 0 (0,0)	<0.001 ***
			Immediately after injection versus during treatment	4 (2,6) 0 (0,2)	<0.001 ***
			Immediately after injection versus at the end of treatment	4 (2,6) 0 (0,0)	<0.001 ***
			During treatment versus at the end of treatment	0 (0,2) 0 (0,0)	0.04 *
2.	WBS	INJEX	Before versus immediately after injection	1 (0,0) 0 (0,2)	0.01 **
			Before versus during treatment	1 (0,0) 0 (0,2)	0.152
			Before versus at the end of treatment	1 (0,0) 0 (0,0)	0.824
			Immediately after injection versus during treatment	0 (0,2) 0 (0,2)	0.135
			Immediately after injection versus at the end of treatment	0 (0,2) 0 (0,0)	0.03 **
			During treatment versus at the end of treatment	0 (0,2) 0 (0,0)	0.182

* Significant at 0.05 level. ** Significant at the 0.01 level. *** Significant at the 0.001 level. WBS = Wong–Baker FACES Pain Rating Scale.

## Data Availability

Data will be provided upon request to the corresponding author.
